# Hematologic Toxicity Profiles and the Impact of Hemoglobin Nadir and Transfusion on Oncologic Outcome in Definitive Radiochemotherapy for Cervical Cancer

**DOI:** 10.3390/cancers16233986

**Published:** 2024-11-27

**Authors:** Eva Meixner, Laura Wermes, Line Hoeltgen, Lisa Antonia von Diest, Elisabetta Sandrini, Semi Harrabi, Katharina Seidensaal, Philipp Hoegen-Saßmannshausen, Maria Vinsensia, Laila König, Nathalie Arians, Jürgen Debus, Juliane Hörner-Rieber

**Affiliations:** 1 Department of Radiation Oncology, Heidelberg University Hospital, Im Neuenheimer Feld 400, 69120 Heidelberg, Germany; laura.wermes@stud.uni-heidelberg.de (L.W.); line.hoeltgen@med.uni-heidelberg.de (L.H.); lisa-antonia.vondiest@med.uni-heidelberg.de (L.A.v.D.); elisabetta.sandrini@med.uni-heidelberg.de (E.S.); semi.harrabi@med.uni-heidelberg.de (S.H.); katharina.seidensaal@med.uni-heidelberg.de (K.S.); philipp.hoegen@med.uni-heidelberg.de (P.H.-S.); maria.vinsensia@med.uni-heidelberg.de (M.V.); laila.koenig@med.uni-heidelberg.de (L.K.); nathalie.arians@med.uni-heidelberg.de (N.A.); juergen.debus@med.uni-heidelberg.de (J.D.); juliane.hoerner-rieber@med.uni-duesseldorf.de (J.H.-R.); 2 Heidelberg Institute of Radiation Oncology (HIRO), 69120 Heidelberg, Germany; 3 National Center for Tumor Diseases (NCT), 69120 Heidelberg, Germany; 4 Heidelberg Ion Therapy Center (HIT), Im Neuenheimer Feld 450, 69120 Heidelberg, Germany; 5 German Cancer Research Center (DKFZ), Clinical Cooperation Unit Radiation Oncology, Im Neuenheimer Feld 280, 69120 Heidelberg, Germany; 6 Department of Radiation Oncology, University Hospital Düsseldorf, 40225 Düsseldorf, Germany

**Keywords:** chemoradiation, anemia, hematotoxicity, cisplatin, lymph node dissection

## Abstract

**Background/Objectives**: Definitive radiochemotherapy with concomitant cisplatin 40 mg/m^2^ weekly represents the standard of care for locally advanced cervical cancer. Current studies (KEYNOTE-A18 and INTERLACE) are intensifying this regimen at the cost of increased hematologic toxicity. We aimed to evaluate influencing factors on hematotoxicity to ensure the safe application of radiochemotherapy. **Methods**: We retrospectively analyzed 147 patients, who received definitive radiochemotherapy between 2000 and 2019 and evaluated laboratory parameters before, after, and at each chemotherapy cycle to evaluate the occurrence, course, and profile of hematotoxicity. Further, we assessed the impact of hemoglobin levels and transfusion on oncological outcomes. **Results**: In a high-risk cervical cancer population with 82.3% of women with FIGO ≥ III stage, the 1-, 2-, and 5-year rates of overall survival (OS) were 89.1%, 74.7%, and 63.3%, and local control (LC) rates were 90.1%, 86.1%, and 75.0%, respectively. Grade 3 leukopenia was present in 2.1% and grade 3 anemia in 4.3%. No higher grade ≥ 4 hematotoxicity was observed. Absolute hemoglobin levels significantly reduced after the fourth cycle, with a median time from the start of therapy to hemoglobin nadir of 36 days. A lower hemoglobin nadir (<9 g/dL) was significantly associated with inferior LC. Red blood cell transfusion was applied in 44.9% of the women; the necessity of transfusion was significantly correlated to inferior OS, LC, and distant control. **Conclusions**: Our results suggest the need for special consideration of increased hematotoxicity and consistent implementation of anemia therapy, particularly from the fourth RT week onwards, to enable full-course definitive radiochemotherapy for locally advanced cervical cancer patients.

## Simple Summary

The occurrence of hematologic toxicity is a crucial side effect during definitive radiochemotherapy with cisplatin weekly for locally advanced cervical cancer and is even challenging with the implementation of current studies with intensified systemic therapy regimens. We aimed to assess the course of toxicity profiles of hematotoxicity. In a high-risk cohort of 147 women with 82.3% FIGO stage ≥ III, our results showed the occurrence of a hemoglobin nadir after the fourth chemotherapy cycle, with a median of 36 days after RT initiation. A value below 9 g/dl was associated with inferior local control, while the necessity of transfusion was significantly correlated to overall survival and distant control. Our analysis suggests the need for special patient monitoring from the fourth week onwards, about 30 days after the start of treatment, to guarantee the safe application of radiochemotherapy.

## 1. Introduction

Cervical cancer is the fourth most common cancer worldwide, with about 600,000 new cases in 2020 and even higher rates of 18.8 per 100,000 in transitioning countries [1]. Definitive radiochemotherapy (RCHT) with concomitant cisplatin 40 mg/m^2^ weekly and magnetic resonance tomography-guided brachytherapy represents the standard of care for locally advanced cervical cancer, achieving 5-year local control rates of 92% [2]. Current studies are intensifying these radiochemotherapies with the addition of concomitant and adjuvant courses of pembrolizumab in the KEYNOTE-A18 study [3] and significantly improved 3-year overall survival (OS) with 82.6% (pembrolizumab) vs. 74.8% (placebo). However, this gain in oncological benefit is accompanied by increased serious hematotoxicity (3% vs. 1%) and treatment-related adverse events, leading to treatment discontinuation due to anemia (2% vs. 1%), neutropenia (2% vs. 1%), neutrophil count decrease (2% vs. 1%), leukopenia (1% vs. 1%), and thrombocytopenia (1% vs. <1%). The INTERLACE study [4] with an additional six cycles of short-course neoadjuvant weekly carboplatin and paclitaxel resulted in improved 5-year OS of 80% (neoadjuvant+ RCHT) vs. 72% (RCHT only) (hazard ratio 0.60). This treatment regimen also challenged the multimodal therapy due to increased grade 3–4 hematological adverse events after neoadjuvant chemotherapy (30%) compared to RCHT alone (13%). The main reason for not completing the planned schedule of six neoadjuvant cycles was neutropenia, and for not reaching five cycles of concomitant cisplatin were hematological reasons in 13.6% (neoadjuvant+RCHT) vs. 1.6% (RCHT only).

As the intensification of therapy performed shortly before or concomitant to standard RCHT goes along with increased side effects, the evaluation of influencing factors on hematological toxicity is important to ensure that definitive radiochemotherapy can be applied safely. However, data on hematotoxicity profiles remain sparse and controversial. We aimed to evaluate the occurrence, course, and profile of hematotoxicity during standard RCHT and the impact of hemoglobin level on oncological outcome. Further, we focused on exploring additional influencing factors on the overall oncological prognosis of cervical cancer patients.

## 2. Materials and Methods

### 2.1. Patient Population and RT Treatment

In this retrospective single-center study, we analyzed patients with locally advanced cervical cancer who received definitive radiochemotherapy at our institution between 2000 and 2019. Data collection and analysis were approved by the local ethics committee (S-453/2021).

Only patients treated with curative intent were included. Patients who were unsuitable for chemotherapy and received RT only, and women with hysterectomy and adjuvant radiochemotherapy or distant metastases (except paraaortic lymph node metastases), were excluded. Further, patients with neoadjuvant chemotherapies and concomitant immunotherapy were excluded. Patients were staged according to the corresponding International Federation of Gynecology and Obstetrics (FIGO) classification 2018 [5] and the eight TNM American Joint Committee on Cancer staging system at diagnosis. Treatment management decisions were determined by a multidisciplinary tumor conference that included gynecologists, radiologists, pathologists, and radiation oncologists.

External photon beam RT (EBRT) was delivered as either intensity-modulated RT (IMRT) or three-dimensional conformal RT (3D-RT) with a total dose of 45–50.4 Gy in 25 to 28 fractions. A simultaneously integrated boost (SIB) was delivered to pelvic or paraaortic lymph node metastases with a dose of 54–58.8 Gy in 25–28 fractions. Clinical target volume (CTV) contouring was performed on 3 mm slice thickness computed-tomography scans with a full bladder, taking into account the tumor extension according to MR images and different states of bladder filling, and including the gross tumor volume, whole cervix, uterus, and pelvic iliac lymph nodes up to the bifurcation, as well as extended field paraaortic lymph nodes in case of infiltration. For the planning target volume (PTV), a margin of 0.5–2 cm was added to the CTV. EBRT was delivered in combination with intracavitary and interstitial high-dose-rate brachytherapy with Iridium-192 with a total dose of 28 Gy in 4 fractions.

### 2.2. Chemotherapy and Hematotoxicity

Chemotherapy was applied concomitantly to EBRT. The preferred regimen was cisplatin 40 mg/m^2^ weekly, with an intended number of five to six cycles. Carboplatin and mitomycin/5-fluorouracil were given as alternatives if patients were intolerant to cisplatin, e.g., due to renal dysfunction. Supportive antiemetic medication and hydration infusion therapy were administered at the discretion of the treating physician. Laboratory values were documented before and after each cycle. Hematotoxicity and peripheral blood cell counts were assessed before and after each cycle and graded according to CTCAE version 5: Anemia (hemoglobin, g/dL): Grade 0: ≥ 11.0, grade 1: 10.9–10.0, grade 2: 9.9–8.0, grade 3: 7.9–6.5, grade 4: <6.5; leukocytopenia (leukocytes,/nL): Grade 0: ≥ 4.0, grade 1: 3.0–3.9, grade 2: 2.0–2.9, grade 3: 1.0–1.9, grade 4: <1.0; and Thrombocytopenia/nL): Grade 0: ≥ 100, grade 1: 75.0–99.9, grade 2: 50.0–74.9, grade 3: 25.0–49.9, grade 4: <25. Analysis of gastrointestinal and urogenital toxicity was not part of this study and will be assessed separately.

### 2.3. Oncologic Outcomes

Clinical outcomes included the assessment of OS, local control (LC), and distant control (DC). OS was defined as the period from the end of RT until the last contact or date of death. LC was considered until any tumor progression at the original site or local pelvic lymph nodes, while DC was defined as no metastatic lesions developing outside the pelvis.

### 2.4. Statistical Analysis

To assess categorical data, Pearson Chi-Square tests were used, while the Mann–Whitney U test and *t*-tests or Wilcoxon signed-rank tests were applied to compare continuous variables. Linear regression was performed to analyze dosimetric factors in uni- and multivariate analyses. A conservative threshold significance level for the *p*-value of less than 0.05 was considered statistically significant for all tests to avoid disregarding interesting correlations or effects that could be investigated in a larger prospective study. ROC (receiver operating characteristic) curves were generated to calculate cut-off values. For statistical calculations, statistical software IBM SPSS (Armonk, NY, USA, version 27) was used.

## 3. Results

### 3.1. Study Population 

Our cohort consisted of 147 women with a median age of 54 years (range: 31–81) with cervical cancer who were treated with definitive radiochemotherapy at our institution between January 2000 and December 2019. Most cervical cancers were staged as FIGO stage III (71.4%), and the most common histologies were squamous cell carcinoma (83.7%), followed by adenocarcinoma (12.2%). Detailed patient and tumor characteristics are listed in Table 1.

The median EBRT dose was 45.0 Gy (range: 34.2–54.0 Gy) in 25 fractions (range: 20–30). A simultaneously integrated boost was delivered in 70 patients (47.6%) with a median dose of 55 Gy (range: 54–58.8 Gy) to the macroscopic nodal disease. Two patients (1.4%) did not complete RT as planned. Brachytherapy was delivered to 146 patients with a median of four fractions (range: 1–6) and a single dose of 7 Gy (range: 5–7 Gy).

One hundred forty patients (95.2%) received cisplatin weekly only, three patients received carboplatin, three patients received mitomycin C/5FU, and the remaining patients received a combination of cis- and carboplatin. RT and chemotherapy characteristics are reported in Table 2.

### 3.2. Oncologic Outcomes

**Figure 1:**
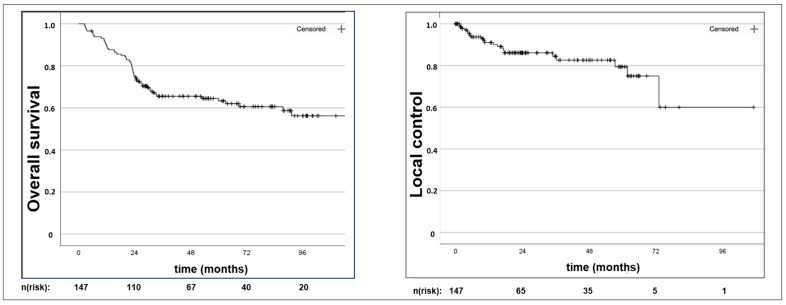
Kaplan–Meier censored survival estimates with overall survival and local control. n(risk): number at risk.

With a median follow-up of 28.9 (range: 0–107) months, the 1-, 2-, and 5-year OS rates were 89.1%, 74.7%, and 63.3%, respectively (Figure 1). Moreover, 21 women suffered from local progression, and 49 patients developed distant metastases, resulting in 1-, 2-, and 5-year LC rates of 90.1%, 86.1%, and 75.0% (Figure 1), and DC rates of 77.4%, 63.3%, and 58.8%, respectively.

Table 3 presents a detailed univariate analysis of potential prognostic factors influencing OS, LC, and DC. In univariate analyses, a higher FIGO and T-stage, a larger lymph node size, the presence of paraaortic lymph node metastases, and the necessity of applying an extended paraaortic RT field were associated with significantly inferior OS. Inferior local control was associated with higher FIGO and T-stages, as well as extended paraaortic RT field application; further, LC was worse in patients with a total treatment duration of more than 8 weeks. Distant control was significantly inferior in patients with higher FIGO stages, the presence of lymph node metastases and paraaortic metastases, larger lymph node sizes, and extended RT fields.

Histology, grading, parametrial infiltration, menopausal status, body mass index, and the number of chemotherapy cycles were not associated with oncological outcomes.

Only a higher T stage (3/4) (*p* = 0.035) and a larger size of lymph node metastases (*p* = 0.017) were confirmed as independent prognostic factors for inferior OS in multivariate analysis. Further, a treatment duration of more than 56 days (*p* = 0.015) was independently associated with worse local control, and a higher FIGO stage (FIGO 3/4) (*p* = 0.008) was significantly correlated with inferior DC in multivariate analysis.

Besides the above-mentioned influence of the size and localization of the lymph node metastases on oncologic outcome, the performance of a surgical lymph node dissection, the time between dissection and start of RCHT, and the presence of extracapsular nodal spread did not have a significant influence on OS, LC, or DC.

**Figure 2:**
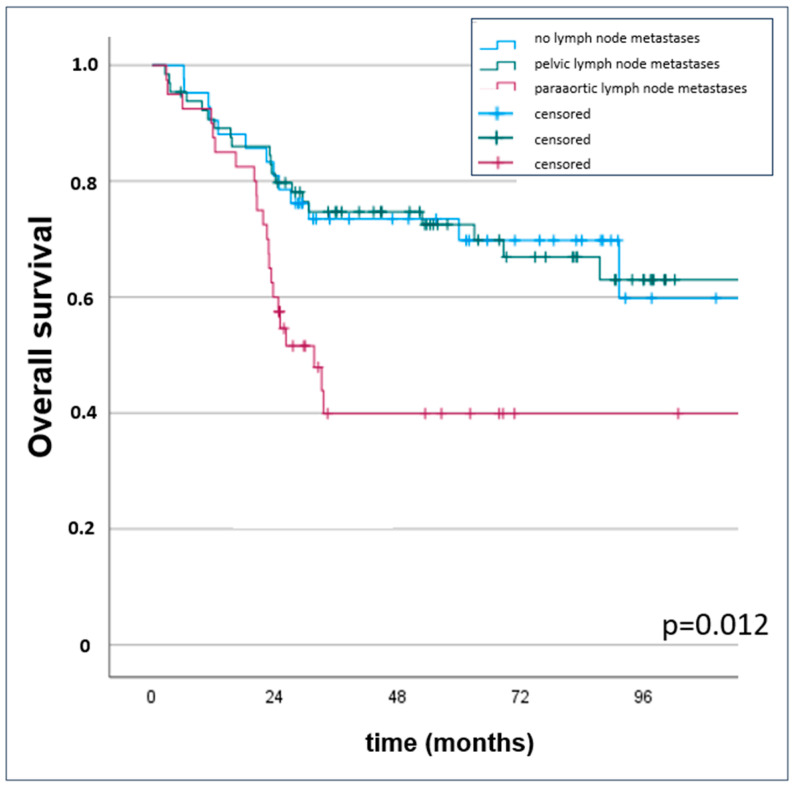
Kaplan–Meier censored survival estimates with overall survival for patients without pelvic and paraaortic lymph node metastases.

Five-year OS was 69.8% for patients with pelvic lymph node metastases, while patients with paraaortic lymph node metastases had a significantly inferior (*p* = 0.012) 5-year OS of 39.9% (Table 3 and Figure 2).

### 3.3. Hematotoxicity and Hemoglobin Levels

**Figure 3:**
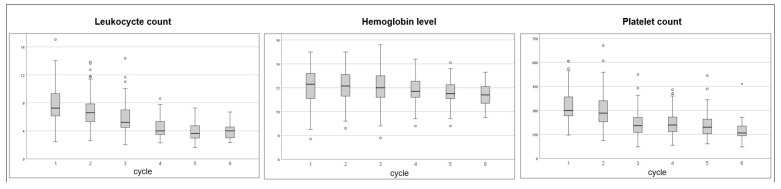
Boxplots with first and third quartiles, outliers, minimum, maximum, and median values of peripheral blood cell counts at the time point of each chemotherapy cycle with leukocytes (per nL; *p* < 0.001 each) and platelet levels (per nL; *p* < 0.001 each) each reducing from the first cycle on and hemoglobin levels (g/dL) significantly reducing after the fourth cycle (*p* = 0.002).

Analyses of hematotoxicity focused on 140 patients who received cisplatin weekly. Figure 3 presents boxplots of peripheral blood counts with leukocyte, hemoglobin, and platelet levels at the time point of each chemotherapy cycle.

The median baseline values were as follows: leukocytes 7.3/nL (range: 2.4–17.1/nL), hemoglobin 12.2 g/dL (range: 7.7–15.0 g/dL), and platelets 296 (range: 146–848/nL).

Absolute leukocyte counts were significantly reduced after each cycle (*p* < 0.001), and leukopenia significantly appeared more often at and after the fourth cycle (*p* < 0.001). Leukopenia was present as grade 1 in 31.4%, grade 2 in 25.7%, and grade 3 in 2.1% of the patients. The median time from RT start until leukocyte nadir was 30 days (range: 4–61).

Absolute hemoglobin levels did significantly reduce after the fourth cycle (*p* = 0.002); anemia appeared significantly more often in the sixth cycle (*p* = 0.008) with grade 1 in 25.0%, grade 2 in 60.7%, and grade 3 in 4.3% of the patients. Sixty-six patients (44.9%) received red blood cell transfusions with a median of one-and-a-half (range: 0–17) transfusions (Table 2).

The median time from the start of RT until hemoglobin nadir was 36 days (range: 0–105); the median time from the start of chemotherapy to hemoglobin nadir was 32 days (range: 0–105).

Platelet counts significantly decreased from the first cycle (*p* < 0.001), while thrombocytopenia was significantly more present in the sixth cycle (*p* = 0.016) with thrombocytopenia grade 1 in 4.3% and grade 2 in 1,4% of the patients. The median time from RT start until thrombocyte nadir was 25 days (range: 1–61). Overall, no higher grade 4 hematotoxicity was documented.

**Figure 4:**
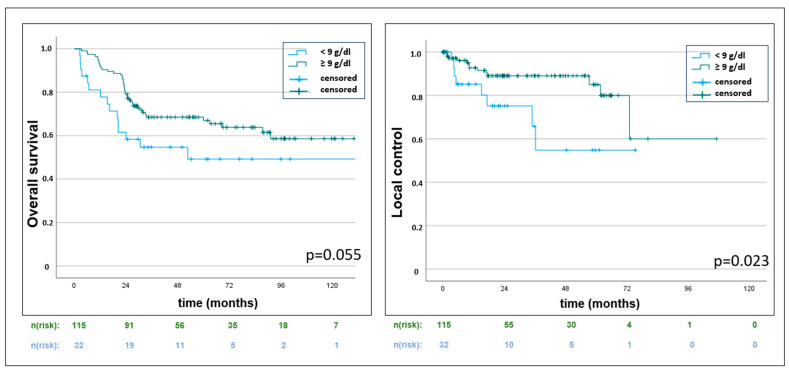
Kaplan–Meier censored survival estimates with a trend for improved overall survival (*p* = 0.055) and statistically significant improved local control (*p* = 0.023) for higher hemoglobin nadir levels of ≥ 9 g/dL versus <9 g/dL.

Higher hemoglobin nadir levels (≥9 g/dL) showed a significantly superior outcome for local control (*p* = 0.023) and a trend for superior OS (*p* = 0.055), the latter without reaching statistical significance (Table 3 and Figure 4). The necessity of red blood cell transfusion was associated with inferior OS, LC, and DC (Table 3).

## 4. Discussion

The avoidance of hematotoxicity is a crucial factor in the definitive treatment of cervical cancer patients. Full knowledge of the course of hematotoxicity in standard RCHT treatment concepts with cisplatin 40 mg/m^2^ weekly is required to ensure safe application.

In our cohort, absolute hemoglobin levels significantly declined after the fourth cycle of chemotherapy, with a median time from the start of therapy to hemoglobin nadir of 36 days. Clinically, this lower hemoglobin nadir (<9 g/dL) resulted in a significantly inferior LC. The necessity of red blood cell transfusion was further significantly correlated to inferior OS, LC, and DC. Grade 3 leukopenia was present in 2.1%, and grade 3 anemia in 4.3%; no higher grade ≥4 hematotoxicity was observed.

The prospective EMBRACE I study [2] reported 5-year OS and LC rates of 75% and 92% in cervical cancer patients treated with definitive RCHT and MRI-guided brachytherapy, while the application of definitive RCHT in our cohort resulted in 5-year OS and LC of only 63.3% and 75%, respectively. However, our study population consisted of a mostly high-risk cervical cancer population, with 82.3% of women staged ≥ FIGO III, while the EMBRACE I study cohort consisted mainly of lower-stage patients, with stage FIGO ≥ III in only 25% of the patients.

Macdonald et al. [6] reported a SEER analysis of 4559 patients with cervical cancer, with 5-year OS rates of 39% for women with paraaortic compared to 67% in patients with pelvic lymph node involvement, which were comparable to our cohort, with 5-year OS rates of 39.9% and 69.8% for the presence of paraaortic and only pelvic lymph node metastases, respectively. In this context, our results confirmed classic prognostic factors and further suggested that the application of a simultaneously integrated boost in node-positive patients apparently seems to make the oncologic outcome comparable to patients without lymph node metastases, irrespective of the performance of a surgical lymph node dissection.

Further, the premature discontinuation or delay of RT and chemotherapy can have a significant impact on prognosis. While only two patients in our cohort did not complete RT as planned due to side effects, an overall treatment duration of more than 56 days was associated with worse local control. However, data on the impact of therapy prolongation remain controversial. Song et al. [7] confirmed treatment time as an independent prognostic factor for pelvic failure, while other studies [8] additionally found a strong correlation to OS. Even if the number of applied chemotherapy cycles was not shown to be a prognostic parameter in our analysis, the results of other studies confirm the impact on prognosis. A cumulative cisplatin dose of more than 200 mg was shown to be associated with superior OS, particularly in patients with lymph node metastases [9], and of more than 250 mg to result in improved 10-year disease-free survival, regardless of weekly or triweekly cisplatin regimens [10].

Considering these results, the application of a full course of chemotherapy to improve outcomes is strongly recommended, but clinical feasibility can be a challenge as the main reasons for not completing the planned schedule of doses are often caused by hematotoxicity. The occurrence of grade 3 to 4 hematological adverse events due to standard radiochemotherapy has been reported in up to 1–26.8% of patients [3,4,11] with even higher incidences in the case of neoadjuvant chemotherapy application [12]. The intensification of therapy regimens with the addition of concomitant and adjuvant courses of pembrolizumab in the KEYNOTE-A18 study [3] significantly improved 3-year OS with 82.6% (pembrolizumab) vs. 74.8% (placebo). However, this benefit is strongly associated with increased serious hematotoxicity (3% vs. 1%) and treatment-related adverse events, leading to treatment discontinuation due to anemia (2% vs. 1%), neutropenia (2% vs. 1%), decreased neutrophil count (2% vs. 1%), leukopenia (1% vs. 1%), and thrombocytopenia (1% vs. <1%). Moreover, short-course neoadjuvant six cycles of weekly carboplatin and paclitaxel in the INTERLACE [4] study improved 5-year OS from 72% (RCHT only) to 80% (neoadjuvant + RCHT), but at the burden of increased grade 3–4 hematological adverse events after neoadjuvant chemotherapy (30%) compared to RCHT alone (13%), consisting mostly of neutropenia (19% vs. 5%). As a consequence, premature neutropenia-caused discontinuation without reaching five cycles of concomitant cisplatin was reported in 13.6% (neoadjuvant+RCHT) vs. 1.6% (RCHT only) of the patients.

However, current studies often only report absolute numbers on the occurrence and grade of hematological adverse events, while the comprehensive toxicity profiles remain unclear. In our cohort, leukocytes, hemoglobin, and thrombocytes reduced significantly after the first, fourth, and first cycles of chemotherapy, respectively, while the median time from RT initiation to leukocyte, hemoglobin, and thrombocyte nadir counts was 30 days, 36 days, and 25 days.

Only a very limited number of studies have previously reported data on the course of blood cell counts. Marinescu et al. [13] retrospectively assessed 69 patients treated with neoadjuvant or concurrent RCHT for cervical cancer and found a slightly later onset of the maximum drop in leukocytes, which was observed before day 35 from the RT initiation. Further, an earlier presentation of the maximum hemoglobin nadir was reported after day 29. Grade ≥ 2 leukopenia, anemia, and thrombocytopenia were further reported to occur after a median time of 29, 31, and 35 days in a cohort of 97 patients with cervical and endometrial cancer by Chen et al. [11]. Overall, these findings, including our results, suggest increased hematotoxicity from the fourth RT week onwards.

In our analysis, women with a hemoglobin nadir level of at least 9 g/dL had significantly superior local control. Baseline values did not seem to have a prognostic impact in our cohort, which is similar to a study by Winter et al. [14], who retrospectively reviewed 494 cervical cancer patients from two prospective Gynecologic Oncology Group (GOG) trials, treated with radiotherapy and cisplatin. Only hemoglobin levels during RCHT predicted treatment outcomes, while pretreatment levels showed no association. However, other studies found a significant difference for progression-free survival (PFS) [15] or even overall survival (OS) [16] with 3-year PFS rates of 73% vs. 71% and 62% depending on pretreatment hemoglobin levels of ≥ 12.0 g/dL, 11.9–10.0 g/dL, or < 10.0 g/dL, respectively [17]. The normalization of pretreatment anemia to normal on-treatment hemoglobin levels was even associated with improved PFS and OS in a study by Koulis et al. [16] with 257 cervical cancer patients treated with chemoradiotherapy.

In our cohort, red blood cell transfusion was applied in almost 45% of the patients, with the necessity of transfusion being significantly correlated to inferior OS, LC, and distant control. But thresholds for triggering transfusion and data regarding the oncologic benefit are contradictory, with some studies favoring different threshold levels of more than 10 g/dL for improved PFS and OS [9] and other studies finding no association between hemoglobin levels or transfusion and outcome at all [18]. Chemotherapy-induced cytopenias can be profound and long-lasting, while there is considerable heterogeneity in the management of hematological toxicity, and current practice patterns, with the optimal timing of erythrocyte transfusion and Granulocyte-colony-stimulating factor (G-CSF) application, remain unclear. While national guidelines favor blood cell transfusion at a level of <10 g/dL [19], some international guidelines only report vague recommendations for monitoring and consensus on correction in case of clinical necessity [20] without a clear threshold. Overall, international expert consensus agreement for transfusion mostly implies a minimum hemoglobin level of at least 9 g/dL [21]. The addition of erythropoietin for the regulation of red blood cell production failed to demonstrate a clinically significant benefit on clinical outcomes in cervical cancer patients [22,23]. The actual implementation in studies is still very heterogeneous and subject to institutional differences. In the INTERLACE trial [4], red blood cell transfusion was permitted and recommended to maintain a hemoglobin level of at least 11 g/dL, while G-CSF was permitted as per standard clinical practice without reporting the frequency of use.

In our cohort, platelet and leukocyte levels decreased from the first cycle on, while hemoglobin levels showed significant reductions at a later stage. This might most likely be explained by the longer life cycle of human red blood cells, which is approximately 120 days [24]. Besides the systemic detrimental effect of chemotherapeutic agents, the release of blood cells from the bone marrow into the blood, and thus the compensation and occurrence of hematotoxicity, might be caused by a variety of other causes, including patient-related factors such as increased body mass index or older age, as well as cytological and immunological factors [13,24]. While overall reduced numbers of blood cells are a common side effect of chemotherapy, particularly with increasing time of exposure, the underlying mechanism of how chemotherapies interfere with healthy hematopoiesis and the influencing factors for persistent or delayed incomplete recovery of the blood cell count are not yet fully understood.

Moreover, treatment-related factors such as the applied RT technique and doses to subvolumes of pelvic bone marrow compartments contributed significantly to the occurrence of hematotoxicity [25,26]. In the RTOG0418 trial [27], improved sparing of bone marrow with IMRT instead of conventional four-field treatments was shown to significantly lower the rates of hematologic toxicity in 43 patients with endometrial and 40 patients with cervical cancer. Chen et al. [11] recommended limiting the dose to functional bone marrow identified by a PET scan, as this had been shown to be significantly associated with the overall occurrence of grade ≥2 hematotoxicity. Machine learning models for grade 4 lymphopenia prediction in cervical cancer patients tried to consider a large number of influencing factors, including patient performance scores, baseline blood counts, the application of concurrent chemotherapy, gross nodal tumor volumes, body volume, and maximum RT doses [28], which resulted in high prediction accuracy. Additionally, analyses on the correlation of hematological biomarkers for outcome prediction have reported platelet-to-lymphocyte ratios [29] and neutrophil-to-lymphocyte [30] ratios to be correlated with oncologic outcome, but data remain inconsistent.

Primary limitations of our study include its retrospective character, which may impact the findings, and the long time interval from 2000 to 2019, with different treatment techniques and possibly different analysis methods of the laboratory parameters. Consequently, our results have to be interpreted cautiously due to potential methodological issues or confounding factors. Further, the applied statistical tests and multiple tests without the application of correction methods may lead to over- or misinterpretation of the results.

However, our cohort consisted of a large, homogenous group of high-risk cervical cancer patients treated with standard radiochemotherapy with cisplatin weekly and provided new data on hematological toxicity profiles and the course of blood counts during treatment. With the increasing intensification of systemic therapies for cervical cancer, knowledge of these processes and hematological courses is an essential requirement for ensuring the safe application of the therapy, particularly recommending close monitoring in the fourth week of treatment and 30 days after RT initiation.

While our study provides a better understanding of the toxicity profiles of acute hematotoxicity during definitive RCHT with cisplatin, the pathophysiology of late or prolonged side effects still remains poorly understood, and randomized trials are needed to assess the optimal management of treating myelosuppression and consecutive transfusion recommendations.

## 5. Conclusions

Hematological toxicity profiles during the course of radiotherapy and concomitant chemotherapy revealed hemoglobin nadir levels and the necessity of transfusion to be significantly correlated with oncologic outcomes. Our results suggest the need for special consideration of increased hematotoxicity and consistent implementation of anemia therapy, particularly from the fourth RT week onwards, to enable full-course definitive radiochemotherapy for locally advanced cervical cancer patients.

## Figures and Tables

**Table 1:** Patient and tumor characteristics.

Characteristics	Value, Percentage, or Range
Median age; years	54 (31–81)
Median body-mass-index	26.5 (16.3–56.8)
Histology	
squamous cell carcinoma	123 (83.7%)
adenocarcinoma	18 (12.2%)
neuroendocrine	2 (1.4%)
mixed	4 (2.7%)
FIGO stage	
I	4 (2.7%)
II	22 (15.0%)
III	105 (71.4%)
IVa	16 (10.9%)
T-stage	
1/2	103 (70.1%)
3/4	44 (29.9%)
Grading	
G1	5 (3.4%)
G2	65 (44.2%)
G3	57 (38.8%)
unknown	20 (13.6%)
Parametrial infiltration	
yes	106 (72.1%)
No	41 (27.9%)
Lymph node metastases	
yes	105 (71.4%)
no	42 (28.6%)
Localization lymph node metastases	
pelvic	65 (61.9%)
pelvic + paraaortic	40 (38.1%)
Size lymph node metastases	
≤10 mm	21 (20.0%)
11–20 mm	59 (56.2%)
>20 mm	24 (22.9%)
unknown	1 (0.9%)
Lymph node dissection performed	
yes	76 (51.7%)
No	71 (48.3%)

**Table 2:** Radiotherapy and chemotherapy characteristics.

Characteristics	Value, Percentage, or Range
Median total dose; Gy	45.0 (34.2–54.0)
Median SIB dose; Gy	55 (54–58.8)
Median treatment duration; days	53 (29–152)
Extended paraaortic RT field	
yes	39 (26.5%)
no	108 (73.5%)
RT technique	
IMRT	138 (93.9%)
3D	9 (6.1%)
Brachytherapy	
yes	146 (99.3%)
no	1 (0.7%)
Chemotherapy	
Cisplatin	140 (95.3%)
Carboplatin	3 (2%)
Cis- and Carboplatin	1 (0.7%)
Mitomycin C/5-FU	3 (2%)
Cisplatin cycles	
≤4	38 (27.1%)
≥5	97 (69.3%)
unclear documentation	5 (3.6%)
Median hemoglobin nadir; g/dL	9.8 (7.5–13.5)
Red blood cell transfusion	
median	1.5 (0–17)
0	81 (55.1%)
2–4	54 (36.7%)
≥5	12 (8.2%)
Median time from lymph node dissection to RT start; days	40 (11–100)

Notably, 3D: three-dimensional conformal radiotherapy, IMRT: intensity-modulated radiotherapy, 5-FU: 5-fluoruracil, RT: radiotherapy, SIB: simultaneously integrated boost.

**Table 3:** Prognostic factors for oncologic outcome in univariate analysis.

Characteristics	Overall Survival		Local Control		Distant Control	
	HR (95%CI)	*p*	HR (95%CI)	*p*	HR (95%CI)	*p*
FIGO stage (1/2 vs. 3/4)	2.04 (1.29–3.25)	0.003	2.90 (1.31–6.40)	0.008	2.18 (1.33–3.56)	0.002
T stage (1/2 vs. 3/4)	1.45 (1.08–1.95)	0.013	1.87 (1.18–2.99)	0.008	1.25 (0.90–1.72)	0.179
Histology (squamous cell carcinoma vs. other)	1.00 (0.71–1.41)	0.986	0.74 (0.31–1.75)	0.492	1.19 (0.89–1.59)	0.239
nodal status (N0 vs. N1)	1.43 (0.77–2.66)	0.246	1.36 (0.45–3.72)	0.549	2.16 (1.05–4.45)	0.037
lymph node size (≤20 mm vs. >20 mm)	1.04 (1.01–1.07)	0.005	1.01 (0.92–1.05)	0.790	1.04 (1.02–1.07)	0.003
localization lymph node (pelvic vs. paraaortic)	1.62 (1.11–2.35)	0.012	1.78 (0.95–3.34)	0.071	1.88 (1.27–2.79)	0.002
lymph node dissection (yes vs. no)	0.98 (0.58–1.67)	0.942	1.11 (0.47–2.61)	0.819	1.40 (0.79–2.46)	0.247
Parametrial infiltration (yes. vs. no)	1.21 (0.65–2.26)	0.543	3.61 (0.84–15.56)	0.085	0.97 (0.52–1.80)	0.922
RT technique (IMRT vs. 3D)	1.55 (0.62–3.88)	0.353	0.05 (0–529.4)	0.518	1.31 (0.32–5.40)	0.709
extended RT field (yes vs. no)	2.50 (1.45–4.33)	0.001	3.03 (1.24–7.43)	0.016	2.51 (1.41–4.55)	0.002
Treatment duration (≤ 56 days vs. >56 days)	1.59 (0.90–2.82)	0.114	2.96 (1.21–7.26)	0.018	1.18 (0.60–2.31)	0.626
Hemoglobin nadir (<9 vs. ≥9 g/dL)	0.56 (0.31–1.01)	0.055	0.356 (0.15–0.87)	0.023	0.64 (0.34–1.21)	0.167
red blood cell transfusion (yes vs. no)	1.16 (1.04–1.29)	0.008	1.28 (1.10–1.50)	0.002	1.20 (1.08–1.33)	0.001

RT: radiotherapy, IMRT: intensity-modulated radiotherapy, 3D: three-dimensional conformal radiotherapy, CI: confidence interval, HR: hazard ratio, *p*: *p*-value.

## Data Availability

The data presented in this study was obtained from local databases of our institution and the Cancer Registry of the National Center for Tumor Diseases (NCT). The data are not publicly available due to Local Ethics Committee rules.
